# Families: the newest members of the ICU multidisciplinary team

**DOI:** 10.1186/cc11102

**Published:** 2012-03-20

**Authors:** R Santhirapala, J Lipton, T Hall, R Breeze, A Molokhia

**Affiliations:** 1 University Hospital Lewisham, London, UK

## Introduction

We have started inviting the relatives of our patients to remain present during our multidisciplinary team ICU ward round. The aim is to improve their understanding of the complex activity on an ICU and reduce inconsistencies in communication. In the UK it is becoming expected practice that patient satisfaction is an endpoint we should be measuring and improving [1]. Assessing this on the ICU is often very difficult due to the confounding factors inherent to critical illness. We often seek assent from families for procedures and to provide some history as a surrogate to patient interview. We think the care we provide should encompass both the patient and their family. This is already accepted practice in the paediatric ICU setting [2]. Communication between family and clinical staff, ideally on a daily basis, is clearly imperative and a systematic approach to improve this is good practice. Increasing insight into relatives' perceptions and expectations will aid the delivery of high-quality care. We believe that involving relatives in the ward round will be of benefit for us in our professional relationships with them and improve their understanding during an extremely difficult time.

## Methods

This was a prospective study over 2 months formally inviting up to four families per day to be present for that part of the ward round involving their relative. Subsequently they were asked to complete a questionnaire anonymously on the experience.

## Results

The results that reflected 31 ward round attendances were unanimous: every family agreed that their attendance had a positive impact, alleviating misconceptions about the intensive care environment and clarifying the processes involved in the care of their relative. The survey also revealed that attendance at the ward round provided an excellent opportunity to have their questions answered by consultants. All those invited wished to attend and all respondents said the experience was valuable and they would like to attend again. Comments included: 'Explanations very helpful to deal with the stress of the situation' and 'Reassuring to have information delivered professionally and compassionately'.

## Conclusion

In this single-centre survey we have demonstrated that inviting families to ICU ward rounds is feasible and we believe that this intervention could improve family satisfaction on the ICU. We are investigating the impact of this intervention with a detailed comparative survey, which we will present in the future.
